# Human Fatal Zaire Ebola Virus Infection Is Associated with an Aberrant Innate Immunity and with Massive Lymphocyte Apoptosis

**DOI:** 10.1371/journal.pntd.0000837

**Published:** 2010-10-05

**Authors:** Nadia Wauquier, Pierre Becquart, Cindy Padilla, Sylvain Baize, Eric M. Leroy

**Affiliations:** 1 Unité des Maladies Virales Émergentes, Centre International de Recherches Médicales de Franceville, Franceville, Gabon; 2 UMR190 Emergence des Pathologies Virales, Université Aix-Marseille II and Institut de Recherche pour le Développement, Marseille, France; 3 Unité de Biologie des Infections Virales Emergentes, Institut Pasteur, IFR128-Biosciences Gerland-Lyon Sud, Lyon, France; University of Texas Medical Branch at Galveston, United States of America

## Abstract

**Background:**

*Ebolavirus* species *Zaire* (ZEBOV) causes highly lethal hemorrhagic fever, resulting in the death of 90% of patients within days. Most information on immune responses to ZEBOV comes from *in vitro* studies and animal models. The paucity of data on human immune responses to this virus is mainly due to the fact that most outbreaks occur in remote areas. Published studies in this setting, based on small numbers of samples and limited panels of immunological markers, have given somewhat different results.

**Methodology/Principal Findings:**

Here, we studied a unique collection of 56 blood samples from 42 nonsurvivors and 14 survivors, obtained during the five outbreaks that occurred between 1996 and 2003 in Gabon and Republic of Congo. Using Luminex technology, we assayed 50 cytokines in all 56 samples and performed phenotypic analyses by flow cytometry. We found that fatal outcome was associated with hypersecretion of numerous proinflammatory cytokines (IL-1β, IL-1RA, IL-6, IL-8, IL-15 and IL-16), chemokines and growth factors (MIP-1α, MIP-1β, MCP-1, M-CSF, MIF, IP-10, GRO-α and eotaxin). Interestingly, no increase of IFNα2 was detected in patients. Furthermore, nonsurvivors were also characterized by very low levels of circulating cytokines produced by T lymphocytes (IL-2, IL-3, IL-4, IL-5, IL-9, IL-13) and by a significant drop of CD3+CD4+ and CD3+CD8+ peripheral cells as well as a high increase in CD95 expression on T lymphocytes.

**Conclusions/Significance:**

This work, the largest study to be conducted to date in humans, showed that fatal outcome is associated with aberrant innate immune responses and with global suppression of adaptive immunity. The innate immune reaction was characterized by a “cytokine storm,” with hypersecretion of numerous proinflammatory cytokines, chemokines and growth factors, and by the noteworthy absence of antiviral IFNα2. Immunosuppression was characterized by very low levels of circulating cytokines produced by T lymphocytes and by massive loss of peripheral CD4 and CD8 lymphocytes, probably through Fas/FasL-mediated apoptosis.

## Author Summary

*Ebolavirus*, especially the species *Zaïre* (ZEBOV), causes a fulminating hemorrhagic fever syndrome resulting in the death of most patients within a few days. *In vitro* studies and animal models have brought some insight as to the immune responses to ZEBOV infection. However, human immune responses have as yet been poorly investigated, mainly due to the fact that most outbreaks occur in remote areas of central Africa. Published studies, based on small numbers of biological samples have given conflicting results. We studied a unique collection of 50 blood samples obtained during five outbreaks that occurred between 1996 and 2003 in Gabon and Republic of Congo. We measured the plasma levels of 50 soluble factors known to be involved in immune responses to viral diseases. For the first time, using a cell staining technique, we analyzed circulating lymphocytes from ZEBOV-infected patients. We found that fatal outcome in humans is associated with aberrant innate immunity characterized by a “cytokine storm,” with hypersecretion of numerous proinflammatory mediators and by the noteworthy absence of antiviral interferon. The adaptive response is globally suppressed, showing a massive loss of CD4 and CD8 lymphocytes and the immune mediators they produce. These findings may have important pathological and therapeutic implications.

## Introduction

Ebolavirus (EBOV) and Marburgvirus (MARV) are among the most deadly human pathogens, causing a severe hemorrhagic fever syndrome in both humans and non human primates [1]–[2]. EBOV is subdivided into five species with different pathogenicities [3]. *Zaire ebolavirus* (ZEBOV), the most lethal species (case-fatality rate up to 90%), caused numerous human outbreaks between 1976 and 2008 in Democratic Republic of Congo, Republic of Congo (RC) and Gabon [4]–[9]. *Sudan ebolavirus* (SEBOV, case-fatality rate about 50%) has caused three documented outbreaks in Sudan and one in Uganda [10]–[14]. *Côte d'Ivoire ebolavirus* (CIEBOV) has been linked to a single, non fatal human case [15]–[16], while the newly discovered *Bundibugyo ebolavirus* (BEBOV) caused an outbreak with a 25% case-fatality rate in 2007 in Uganda [17]. Finally, *Reston ebolavirus* (REBOV), which has caused outbreaks in non human primates and swine in the Philippines, appears to be non pathogenic for humans [18]–[20].

EBOV and MARV initially replicate massively in macrophages and dendritic cells (DC), then spread rapidly to all vital organs, infecting endothelial cells, epithelial cells, hepatocytes, and other cell types [21]–[25]. Infection by MARV and the most lethal EBOV species, described virtually exclusively *in vitro* and with experimental animal models, is associated with high-level viremia, abundant proinflammatory cytokine and chemokine production, massive bystander lymphocyte apoptosis, and widespread focal tissue destruction, resulting in increased endothelial cell permeability, multiorgan failure, and severe clotting disorders, and culminating in a final septic shock-like syndrome [26]–[29]. Fatal outcome in experimentally infected mice and non human primates is associated with impairment of innate immune responses, including rapid and important secretion of certain inflammatory mediators and the absence of type I interferon (IFN) production, and also with profound suppression of adaptive immune responses, including impaired humoral responses and B and T lymphocyte apoptosis [30]–[35].

Most information on filovirus pathogenesis comes from *in vitro* studies and experimental models. However, experimentally infected animal models fail to reproduce certain features of human ZEBOV infection. For instance, rodent models do not exhibit hemorrhagic manifestations and often fail to develop disseminated intravascular coagulation. Lymphocyte apoptosis is consistently observed in mice infected with an adapted variant of ZEBOV but has not been reported in ZEBOV-infected guinea pigs despite histological evidence in the spleen or lymphoid organs of fatally infected animals [26], [36]–[38]. Non human primate models best mimic fatal human infection, but they do not reproduce the survival of a small percentage of patients [39]–[43]. The paucity of data on human immune responses to ZEBOV is largely due to the fact that most outbreaks occur in remote areas where the facilities required to handle and process clinical samples are lacking. Only four studies of human filovirus infection have been reported so far, only two of which focused on ZEBOV. These two studies were small, involved few immunological markers, and gave conflicting results. The first study involved 9 patients infected during the 1995 Kikwit outbreak (7 fatal cases and 2 survivors), and showed slightly higher serum levels of IFN-γ, IFN-α, TNF-α, IL-2 and IL-6 in the non survivors than in the survivors, suggesting that stronger immune activation was associated with fatal outcome [44]. The second study, involving 12 patients infected during the 1996 Gabon outbreaks (8 fatal cases and 4 survivors), failed to confirm the link between elevated IFN-α, TNF-α or IL-2 secretion and fatal outcome [45]–[48]. This latter study suggested that fatal outcome was associated with generalized immune suppression, including intravascular apoptosis, a lack of ZEBOV-specific IgG production, and defective early inflammatory responses when compared to non fatal and asymptomatic infection. However, evidence of lymphocyte apoptosis was based on DNA fragmentation in peripheral blood mononuclear cells (PBMC) and reduced CD3, CD8, IFN-γ, IL-2 and IL-4 mRNA levels, which cannot distinguish apoptosis from necrosis or anergy, or identify the different target cell subsets. Innate immunity has only been investigated in 8 fatal cases and 4 survivors. Because of the known variability of human immune responses to a given pathogen, and differences in immune status at the time of infection, due for example to concomitant infections by other pathogens, larger studies are needed to observe statistically meaningful trends.

To further characterize human immune responses during the acute phase of ZEBOV infection, we analyzed a unique collection of 56 blood samples collected during the five outbreaks that occurred between 1996 and 2003 in Gabon and RC.

## Materials and Methods

### Ethics Statement

This study was implemented as part of an Ebola outbreak control operation coordinated by Ministries of Health (MoH) of Gabon and RC, and therefore no ethics committee approval was deemed necessary, as it is usually the case in this kind of emergencies. The patients described here are anonymous. Blood samples were collected by a team from CIRMF (Centre International de Recherches Médicales de Franceville), Gabon, participating in the international response to the different outbreaks along with healthcare workers from MoH of Gabon and RC, the World Health Organization (WHO), Médecins sans Frontières, the Centers for Disease Control and Prevention (CDC), Atlanta, USA, and the National Microbiology Laboratory, Winnipeg, Canada. Blood samples were collected at the patient's home or in hospital isolation wards, with WHO and MoH authorizations (File S1 and File S2), and with verbal consent from the patient. The two study protocols were reviewed and approved together by the scientific committee of CIRMF.

### Outbreaks and patients

All suspected cases, identified by the international medical teams, were isolated, sampled and received symptomatic treatment and palliative care. Blood samples were collected during the acute phase from patients with laboratory-confirmed ZEBOV infection, during all the documented ZEBOV outbreaks that occurred in Gabon and RC between 1996 and 2005. The first outbreak hit the villages of Mayibout I and II, located in north-eastern Gabon, from January to February 1996, causing 10 non fatal clinical cases and 21 deaths. The second outbreak caused 45 deaths among 60 cases between October 1996 and March 1997 in the Booué area, ∼150 km southwest of Mayibout. The infection spread to several villages around Booué, then to Libreville, where 15 cases were recorded. The third outbreak occurred between October 2001 and May 2002 in the Mekambo area of Gabon and the Mbomo area of RC, ∼150 km east of Mayibout. This outbreak in fact consisted of several independent epidemic chains of human transmission that arose from infected animal carcasses (mainly chimpanzees and gorillas). A total of 207 human cases (58 survivors and 149 deaths) were recorded. There were 15 survivors and 128 deaths recorded during the third outbreak which again affected the region of Mbomo in RC, between December 2002 and May 2003. This outbreak had two independent sources, both due to handling of animal carcasses, one in Yembelengoye village and one in Mvoula, a gold-digger camp located further east, and mainly affected Mbomo and Kelle. Finally, the last outbreak affected the region of Mbomo, causing 6 deaths among 35 cases between October and December 2003. Initial cases occurred in Mbanza, a village located about 30 km north of Mbomo.

### Biological samples

Upon collection, blood samples were immediately transported to CIRMF. Plasma and sera were separated by centrifugation and stored at −80°C until use. When enough blood was available, PBMC were separated from whole blood by density gradient centrifugation on lymphocyte separation medium (Eurobio) at 2300 rpm for 20 min at room temperature, then washed with phosphate buffered saline (PBS)/2% fetal calf serum (FCS), and cryopreserved in FCS containing 10% DMSO in liquid nitrogen in CIRMF secure facilities.

Thirty control plasma samples were randomly selected among 4,349 samples collected from healthy individuals throughout Gabon during a previous study [49]. These individuals were themselves randomly selected among the Gabonese rural population excluding children and elderly persons (more than 65 years). All controls were native Gabonese.

ZEBOV infection was confirmed by detection of circulating antigens with reagents kindly provided by the CDC, Atlanta.

### Assays of circulating cytokines, chemokines and growth factors

Levels of 50 cytokines, chemokines and growth factors were measured in plasma samples by using Luminex technology (Bio-Rad). Two kits, the Bio-plex human cytokine 27-plex assay and the Bio-plex human cytokine 23-plex assay (Bio-Rad), were used as recommended by the manufacturer. The target cytokines were interleukin-1β (IL-1β), IL-1 receptor antagonist (IL-1RA), IL-2, IL-4, IL-5, IL-6, IL-7, IL-8, IL-9, IL-10, IL-12p70, IL-13, IL-15, IL-17, eotaxin, basic fibroblast growth factor (FGF-basic), granulocyte colony-stimulating factor (G-CSF), granulocyte macrophage colony-stimulating factor (GM-CSF), IFN-γ, IFN-inducible protein 10 (IP-10), monocyte chemoattractant protein-1 (MCP-1), macrophage inflammatory protein-1α (MIP-1α), MIP-1β, platelet-derived growth factor-ββ (PDGF-ββ), regulated-on-activation normal T-cell expressed and secreted (RANTES), tumor necrosis factor-α (TNF-α), and vascular endothelial growth factor (VEGF) for the 27-plex assay; and Il-1α, IL-2Rα, Il-3, Il-12p40, IL-16, IL-18, cutaneous T cell attracting chemokine (CTACK or CCL27), growth regulated oncogene alpha (GRO-alpha or CXCL1), hepatocyte growth factor (HGF), intracellular adhesion molecule 1 (ICAM-1), IFN-α2, leukemia inhibitory factor (LIF), MCP-3 (or CCL7), macrophage colony-stimulating factor (M-CSF), monokine induced by interferon-gamma (MIG or CXCL9), nerve growth factor-β (NGF-β), stem cell factor (SCF), stem cell growth factor-β (SCGF-β), SDF-1α (or CXCL12), tumor necrosis factor-β (TNF-β), TNF-related-apoptosis-induced-ligand (TRAIL) and vascular cell adhesion molecule 1 (VCAM-1) for the 23-plex assay. Briefly, 25 µL of plasma was diluted 1∶4 and incubated with anti-cytokine antibody-coupled beads for 1 h. All incubations were performed at room temperature. Between each step, the complexes were washed three times in wash buffer (Bio-Rad) using a vacuum manifold. The beads were then incubated with a biotinylated detector antibody for 1 hour, before incubation with streptavidin-phycoerythrin for 30 min. Finally, the complexes were resuspended in 125 µL of detection buffer and 200 beads were counted during acquisition in the Luminex 200 device (Bio-Rad). Mean fluorescence intensity was used to calculate final concentrations in pg/mL.

### PBMC phenotyping

Cryopreserved PBMC were rapidly thawed in a 37°C water bath, washed three times and incubated overnight at 37°C in RPMI 1640 culture medium (Life Technologies, UK) with 10% heat-inactivated FCS (full RPMI-10% FCS), 1% penicillin-streptomycin, 1% non essential amino acids, and 1 M HEPES. The cells were then washed in RPMI medium, adjusted to a density of 1×10^6^ cells/mL, and cultured for 18 hours before harvesting and a further wash in RPMI. Approximately 1×10^6^ cells were labeled for 20 min at room temperature with anti CD3-FITC, CD4-PE, CD8-PC7 and CD95-PC5 antibodies (Beckman-Coulter, Geneva, Switzerland). The cells were washed and resuspended in PBS 2% FCS, then 100,000 events were analyzed with an FC500 four-color flow cytometer (Beckman Coulter). Results were analyzed with CXP software (Beckman Coulter). PBMC from three healthy individuals living in rural areas of Gabon who were sampled at the time of the outbreak served as controls.

### Statistical analysis

Student's *t* test or the Mann-Whitney-Wilcoxon test was used to compare values in patient groups and controls. STATA software version 9.0 (Stata Corporation, College Station, USA) was used, and statistical significance was assumed when p<0.05.

## Results

### Levels of circulating cytokines, chemokines and growth factors

The patients were subdivided according to clinical outcome (survivors, S, and deceased, D) and the number of days between symptom onset and sampling (early, S1 and D1; late, S2 and D2). S1 and D1 samples were collected 1–4 days after symptom onset; S2 and D2 were collected ≥5 days after symptom onset (Table S1). Given that disease course in all fatal cases lasted 6–7 days, D2 group contained patients in their last 2–3 days before death.

Levels of the following soluble mediators did not differ significantly between the patient population (S1, S2, D1, D2) and the controls: IFN-α2, IFN-γ, IL-7, IL-12p40, IL-12p70, IL-17, IL-18, TNF-α, TNF-β, TRAIL, FGF-basic, LIF, MIG, MIP-1α, MCP-3, SDF-1α, IL-2rα, G-CSF, GM-CSF, VEGF, PDGF-ββ, SCGF-β, ICAM1, VCAM1, RANTES, IL-1α, HGF, β-NGF, SCF and CTACK (Figure S1).

**Figure 1:**
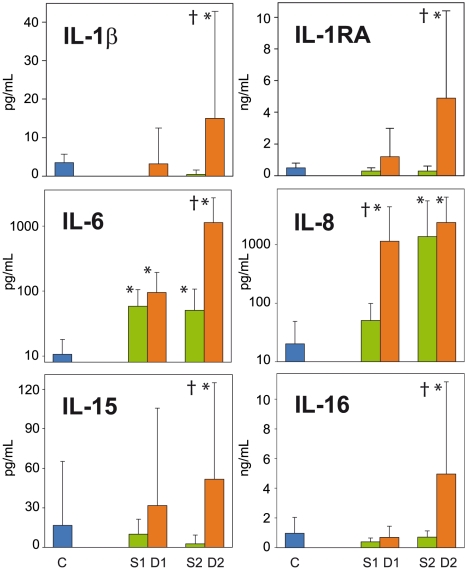
Circulating proinflammatory cytokines upregulated in fatal (red plots, D) and non fatal (green plots, S) cases of clinical ZEBOV infection. Fatal and non fatal cases were each subdivided into two groups according to the interval between symptom onset and blood sampling, as follows: S1 and D1 sampled 1–4 days after symptom onset, S2 and D2 sampled ≥5 days after symptom onset. Given that disease course in all fatal cases lasted 6–7 days, D2 group contains patients sampled in the last 2–3 days before death. Cytokine levels were compared with those found in 30 randomly selected healthy volunteers (blue plots). Results are shown as mean values in each group, and the bars on the plots indicate the standard errors. Asterisks (*) indicate statistically significant differences between patients and healthy controls (p<0.05). Symbols † indicate statistically significant differences between survivors and fatal cases (p<0.05).

**Figure 2:**
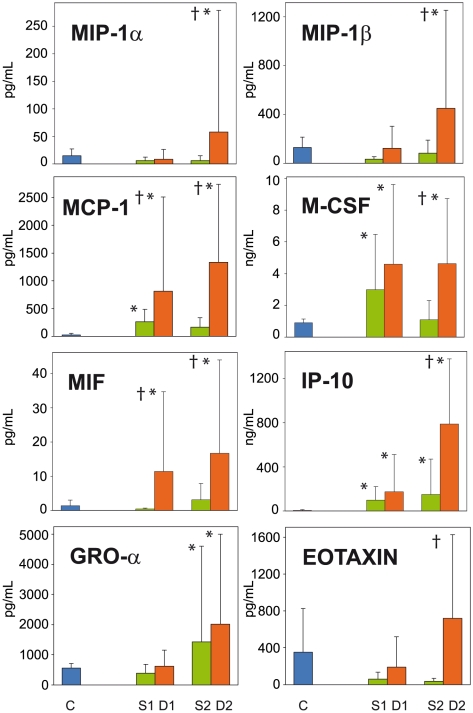
Upregulated circulating chemokines in fatal (red plots, D) and non fatal (green plots, S) cases of clinical ZEBOV infection.

**Figure 3:**
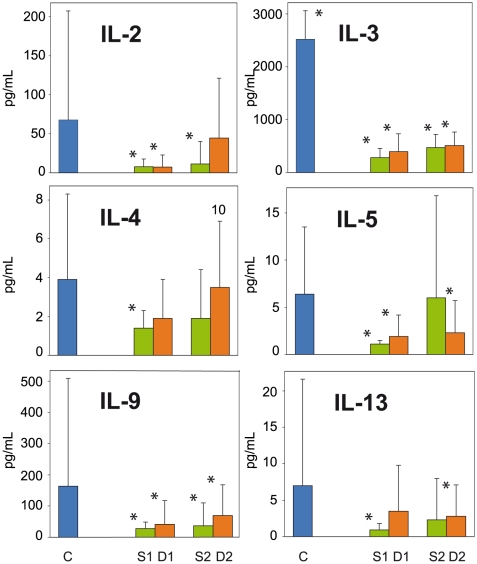
Downregulated cytokines mainly secreted by T lymphocytes, in fatal (red plots, D) and non fatal (green plots, S) cases of clinical ZEBOV infection.

The only significant differences between S1/D1 and controls were higher levels of IL-6, IL-8, MCP-1, M-CSF, MIF (only D1) and IP-10 in the patients (p<0.05) (Figures 1 and 2), while the only significant differences between S1 and D1 were higher IL-8, MCP-1, and MIF levels in non survivors than in survivors (p<0.05) (Figures 1, 2 and 3).

Somewhat surprisingly, the only significant differences between S1 and S2 were higher values of IL-8, MIF and GRO-α in the later samples (p<0.05).

By contrast, marked differences in the levels of several cytokines, chemokines and growth factors were observed between D1 and D2. We also found significant differences between the control samples and D2, and between D2 and S2. Circulating levels of the inflammatory cytokines IL-1β, IL-1RA, IL-6 and IL-16 were significantly (p<0.05) higher in D2 samples than in controls, S1, S2 and D1 samples (Figure 1). Levels in D2 were higher than S1 or controls for IL-8 and IL-15. Levels of all these cytokines were between 5 and 1,000 times higher in D2 samples than in controls. D2 samples contained very high levels of IL-1RA (mean 4.8 ng/mL, SD 5.5 ng/mL; 10 times the control value), IL-6 (mean 1.2 ng/mL, SD 1.6 ng/mL; 100 times the control value), and IL-8 (mean 2.4 ng/mL, SD 4.2 ng/mL; 1000 times the control value) (Figure 1). Similarly, levels of the chemokines MIP-1α, MIP-1β, MIF, IP-10, GRO-α and eotaxin were significantly (p<0.05) higher in D2 samples than in controls, S1, S2 and D1 samples (Figure 2). Levels in D2 were higher than S2 or controls for MCP-1 and M-CSF. Again, levels of these chemokines were between 5 and 1,000 times higher in D2 samples than in controls. The following two chemokines were found at very high levels in D2 samples: MCP-1 (mean 1.3 ng/mL, SD 1.4 ng/mL; 500 times the control value) and IP-10 (mean 7.9 ng/mL, SD 5.9 ng/mL; 1000 times the control value).

Levels of circulating cytokines mainly produced by T lymphocytes (IL-2, IL-3, IL-4, IL-5, IL-9, IL-13) were either similar or significantly lower (p<0.05) in surviving and in non surviving patients than in the controls, especially in early samples (Figure 3).

Levels of cytokines did not differ significantly between different outbreaks, ruling out any temporal bias (Data not shown).

### PBMC phenotyping results

The samples used in this part of the study, including those from healthy individuals, were all obtained during the 2001 ZEBOV outbreak in Gabon.

Frozen PBMC from two healthy controls, three patients sampled 0–1 days before death, one survivor sampled 5 days after symptom onset, and three survivors sampled two weeks after recovery were analyzed by flow cytometry. Positive gating for lymphocytes based on forward and side scatter was followed by CD3+CD4+ and CD3+CD8+ gating, and specific populations were further defined by using antibodies specific for CD95. The results are reported as the percentage of PBMC found in each gate.

**Figure 4:**
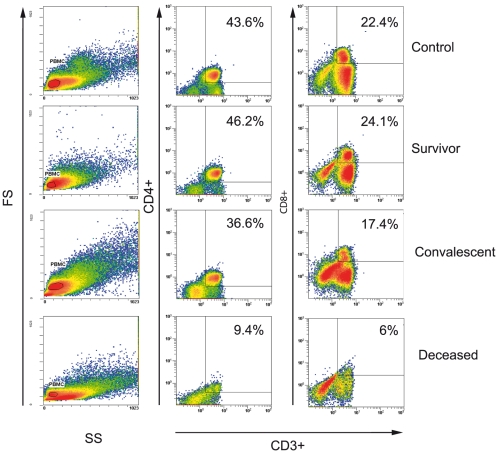
Flow cytometry of PBMC (CD3, CD4 and CD8 phenotyping) collected from fatal and non fatal cases of clinical ZEBOV infection, and from convalescent sampled two weeks after recovery. Results are presented as individual pictures (one individual per group). Mean percentages of gated cells (side and forward scatter) in each group are shown on each picture.

As expected, the percentages of CD3+CD4+ and CD3+CD8+ cells in the two healthy controls were normal (respectively 43.6% and 22.4%) and similar to those both in the survivor sampled during the acute phase (46.2% and 24.1% respectively) and the three survivors sampled after recovery (mean: 36.6% and 17.4%, respectively, Figure 4). By contrast, the percentages of these two lymphocyte populations in the three fatally infected patients were drastically lower than in the controls and survivors: 9.4% CD3+CD4+ cells and 6% CD3+CD8+ cells (Figure 4).

**Figure 5:**
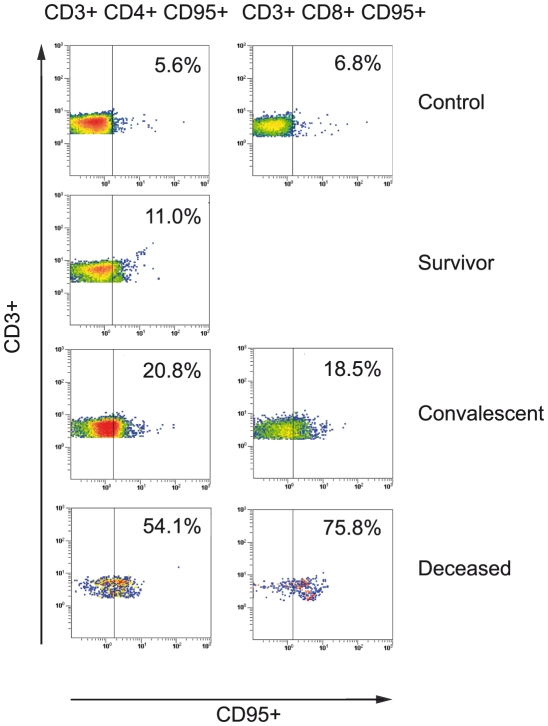
Flow cytometry of PBMC (CD3, CD4, CD8 and CD95 phenotyping) collected from fatal and non fatal cases of clinical ZEBOV infection, and from convalescent sampled two weeks after recovery. Results are presented as individual pictures (one individual per group). Mean percentages of gated cells (side and forward scatter) in each group are shown on each picture.

These data were compatible with the massive lymphocyte death observed elsewhere in experimentally infected animals and *in vitro*. In order to identify the underlying mechanisms during human ZEBOV infection, we determined the percentages of CD3+CD4+ and CD3+CD8+ cells also expressing CD95 (Fas), a specific surface marker of apoptosis. CD3+CD4+CD95+ and CD3+CD8+CD95+ cells represented respectively 54.1% and 75.8% of PBMC in the three ZEBOV fatalities, compared to 5.6% and 6.8% in the two healthy individuals (Figure 5). The percentage of CD3+CD4+CD95+ cells in the survivor sampled during the acute phase of ZEBOV infection was 11%, while the mean percentages of CD3+CD4+CD95+ and CD3+CD8+CD95+ cells in the three post-recovery samples were 20.8% and 18.5%, respectively (Figure 5).

## Discussion

This study, the largest to date, shows that human fatal ZEBOV infection is associated with a markedly impaired innate immune reaction, characterized by strong proinflammatory cytokine production, undetectable antiviral IFNα, and profound immunosuppression resulting from massive peripheral T lymphocyte apoptosis mediated probably in great part by Fas/FasL interactions.

Non survivors had extremely high circulating levels of numerous proinflammatory cytokines (IL-1β, IL-1RA, IL-6, IL-8, IL-15 and IL-16), as well as chemokines and growth factors (MIP-1α, MIP-1β, MCP-1, M-CSF, MIF, IP-10, GRO-α and eotaxin). Levels of these mediators rose rapidly after symptom onset in non survivors, reaching very high levels in the two days before death and creating a ‘cytokine storm’: shortly before death, average levels were between 5 and 1,000 times higher (even more in some individuals) than those observed in both healthy individuals and survivors.

Proinflammatory cytokines, chemokines and growth factors are mainly synthesized by monocytes and dendritic cells and represent the cornerstone of the innate immune reaction to pathogens. At moderately elevated concentrations, these soluble mediators act at various points in the first line of defense, recruiting circulating mononuclear cells to the site of infection, increasing endothelial permeability, activating macrophage and DC cytotoxic functions, and inducing adaptive immune responses by providing co-stimulatory signals for naïve T cells. By contrast, we never detected a raise in IFN-α2 in either survivors or non survivors, suggesting that direct antiviral activity is lacking in ZEBOV-infected patients.

Some of these results are consistent with those of *in vitro* studies and animal models. Indeed, macrophages challenged with EBOV and MARV *in vitro* release large quantities of several proinflammatory cytokines and chemokines, while production of type I IFNs is inhibited. Dendritic cells (DCs), on the other hand, fail to produce cytokines when infected [23], [25], [50]–[52]. Similarly, fatal outcome in experimentally infected mice and non human primates is associated with impairment of innate immune responses, including rapid and important secretion of inflammatory mediators, contrasting with the absence of type I interferon production [30]–[35].

Numerous studies have shown that VP35 and VP24 play an essential role in the ZEBOV suppression of IFN-α/β production and/or response by infected DCs and macrophages [53]–[57]. VP24 interrupts nuclear accumulation of tyrosine-phosphorylated STAT1 and STAT2 in infected cells, making them insensitive to IFN-α/β [58]–[59]. VP35 inhibits phosphorylation, activation and nuclear localization of the interferon regulatory factors 3 and 7 (IRF-3 and IRF-7), transcription factors crucial for IFN-α/β synthesis [60]–[65]. VP35 is also reported to inhibit activation of dsRNA-binding protein kinase (PKR) and the RNAi pathway, again antagonizing the interferon response [66]–[67].

The second remarkable finding of this study is that human fatal ZEBOV infection is associated with a lack of adaptive immunity, reflected by very low levels of circulating cytokines produced by T lymphocytes and by massive loss of CD4 and CD8 lymphocytes. Using Luminex technology, we found that levels of numerous circulating T cell cytokines (IL-2, IL-3, IL-4, IL-5, IL-9, IL-13) were much lower in non survivors than in healthy individuals. Furthermore, using cytometry analysis, we found that CD4 and CD8 lymphocytes represented only 9.2% and 6%, respectively, of PBMC in ZEBOV fatalities, compared to more than 40% and 20% in healthy individuals and survivors. Respectively 54.1% and 75.8% of these cells expressed CD95, values about 10 times higher than those observed in the healthy individuals. These findings, although they are based on a small sample size, confirm and extend the results of the only previous study in natural human ZEBOV infection, which showed marked DNA laddering of PBMC and release of the apoptotic 41/7 NMP protein in ZEBOV fatalities [45], [68]. This latter study did not specify which PBMC subsets underwent apoptosis, or provide information on the underlying mechanism. We found that T CD4 and CD8 lymphocytes underwent massive apoptosis in ZEBOV fatalities, largely through Fas/FasL interaction, whereas the level of lymphocyte apoptosis seen in the survivors was close to that found in the healthy controls. These findings are consistent with the marked bystander lymphocyte apoptosis associated with fatal ZEBOV infection in experimental animals. Studies using flow cytometry, electron microscopy and TUNEL staining have shown that NK, CD4 and CD8 T cells are markedly depleted both through classical apoptosis and through apoptosis-like programmed cell death in the blood and spleen of ZEBOV-challenged BALB/c mice [69]–[70]. Similarly, using the same *in situ* techniques as those mentioned above, recent studies have shown that lymphocytes undergo massive apoptosis in the spleen and lymph nodes of experimentally infected non human primates [24], [30], [33]. In addition, ZEBOV infection of human PBMC *in vitro* has been shown to induce apoptosis of CD4 and CD8 T lymphocytes [71].

Our findings, together with those of *in vitro* studies and animal models, indicate that lymphocyte apoptosis may account for the lymphopenia and lymphoid depletion associated with ZEBOV infection. This lymphocyte apoptosis is likely to involve several pathways, but we show that apoptosis via Fas/FasL interaction is largely responsible for the massive lymphocyte death observed in human fatal ZEBOV infection. This is consistent with *in vitro* studies and experimental ZEBOV infection of macaque monkeys, showing that Fas/FasL and, to a lesser extent TNF-TRAIL mechanisms, may largely account for lymphocyte apoptosis in this setting. Infection of naïve PBMC with ZEBOV *in vitro* strongly upregulates Fas/FasL expression on CD4 and CD8 T lymphocytes and also TNF-related apoptosis-inducing ligand (TRAIL) mRNA expression in the same cells [71]. Similarly, TRAIL and Fas transcript levels were shown to be transiently increased in ZEBOV-infected cynomolgus monkeys [24], [32]. Alternatively, DC dysfunction may lead to bystander lymphocyte apoptosis. Dendritic cells, and to a lesser extent macrophages, play a pivotal role in both innate and adaptive immunity to many viruses. First, these cells secrete antiviral type I IFNs (mainly IFN-α/β) and also proinflammatory cytokines and chemokines that upregulate and guide the adaptive immune response to express specific functions. Second, DCs initiate adaptive immune responses by presenting antigens to T lymphocytes and by stimulating T and B cell differentiation. Thus, early productive replication of EBOV and MARV in macrophages and DCs is likely to impair both innate and adaptive immune responses. The soluble apoptotic factor nitric oxide (NO), synthesized by infected macrophages, as well as the apoptosis-inducing ligands FasL and TRAIL, and immunosuppressive sequences in the viral glycoprotein, have also been implicated in lymphocyte apoptosis in this setting [32], [71]–[73]. Another possibility is that marked DC functional impairment may induce an overall immunosuppressive state. Indeed, several *in vitro* studies have shown that EBOV and MARV infection of DCs fails to activate these cells, thereby inducing altered cytokine expression and interfering with the ability of DCs to express co-stimulatory molecules [24]–[25], [33], [52]. Such DC functional impairment is thought to reduce T cell stimulatory activity and to abrogate functional adaptive immune responses.

This work shows that fatal outcome is associated with aberrant innate immune responses and global suppression of adaptive immunity. The innate response in non survivors leads to a “cytokine storm” which probably rapidly triggers disseminated intravascular coagulation, vascular dysfunction and hypotension and, together with massive lymphocyte apoptosis, likely contributes to vascular collapse, multiple organ failure and the shock-like syndrome associated with human fatal ZEBOV infection.

## Supporting Information

Figure S1 Detection of six unmodified circulating inflammatory cytokines in fatal (red plots, D) and nonfatal (green plots, S) clinical cases of ZEBOV infection. Fatal and nonfatal cases were each subdivided into two groups according to the interval between symptom onset and blood sampling, as follows: S1 and D1 sampled 1–4 days after symptom onset, S2 and D2 sampled ≥5 days after symptom onset. Given that disease course in all fatal cases lasted 6–7 days, D2 group contains patients sampled in the last 2–3 days before death. Cytokine levels were compared with those found in 30 randomly selected healthy volunteers (blue plots). Results are shown as mean values in each group, and the bars on the plots indicate the standard errors.
(0.85 MB EPS)

Table S1 Numbers of healthy individuals and survivors and nonsurvivors of clinical ZEBOV infection. Fatal and nonfatal cases were each subdivided into two groups according to the interval between symptom onset and blood sampling, as follows: S1 and D1 sampled 1–4 days after symptom onset, S2 and D2 sampled ≥5 days after symptom onset. Given that disease course in all fatal cases lasted 6–7 days, D2 group contains patients sampled in the last 2–3 days before death.
(0.03 MB DOC)
