# Supplementary material for: Human Fatal Zaire Ebola Virus Infection Is Associated with an Aberrant Innate Immunity and with Massive Lymphocyte Apoptosis
Source: PLoS Negl Trop Dis. 2010 Oct 5;4(10):e837. doi: 10.1371/journal.pntd.0000837 (PMC2950153; doi:10.1371/journal.pntd.0000837)
Supplement: Table S1 — Numbers of healthy individuals and survivors and nonsurvivors of clinical ZEBOV infection. Fatal and nonfatal cases were each subdivided into two groups according to the interval between symptom onset and blood sampling, as follows: S1 and D1 sampled 1–4 days after symptom onset, S2 and D2 sampled ≥5 days after symptom onset. Given that disease course in all fatal cases lasted 6–7 days, D2 group contains patients sampled in the last 2–3 days before death. (0.03 MB DOC) [file pntd.0000837.s002.doc]

**Table 1**

|  | Early samples (1) | Late samples (2) | Total |
| --- | --- | --- | --- |
| Healthy individuals |  |  | 30 |
| Non fatal cases (S) | S1=4 | S2=10 | 14 |
| Fatal cases (D) | D1=16 | D2=26 | 42 |
